# Genomic investigation of *Salmonella enterica* Serovar Welikade from a pediatric diarrhea case first time in Shanghai, China

**DOI:** 10.1186/s12864-024-10489-7

**Published:** 2024-06-17

**Authors:** Yinfang Shen, Yibin Zhou, Jingyu Gong, Gang Li, Yue Liu, Xuebin Xu, Mingliang Chen

**Affiliations:** 1 Department of Pediatrics, Meilong Community Health Center of Minhang District, Shanghai, China; 2 Department of Infectious Disease Control, Center for Disease Control and Prevention of Minhang District, Shanghai, China; 3 Jinshan Hospital, Fudan University, Shanghai, China; 4 Department of Microbiology, Shanghai Municipal Center for Disease Control and Prevention, Shanghai, China; 5 Research and Translational Laboratory of Acute Injury and Secondary Infection, and, Department of Laboratory Medicine , Minhang Hospital, Fudan University, Shanghai, China

**Keywords:** *Salmonella* Welikade, Foodborne transmission, Diarrhea, Whole genome sequencing, Virulence gene

## Abstract

**Background:**

*Salmonella*, an important foodborne pathogen, was estimated to be responsible for 95.1 million cases and 50,771 deaths worldwide. Sixteen serovars were responsible for approximately 80% of *Salmonella* infections in humans in China, and infections caused by a few uncommon serovars have been reported in recent years, though not with *S.* Welikade. This study reports the first clinical case caused by *S.* Welikade in China and places Chinese *S.* Welikade isolates in the context of global isolates via genomic analysis. For comparison, *S.* Welikade isolates were also screened in the Chinese Local Surveillance System for *Salmonella* (CLSSS). The minimum inhibitory concentrations (MICs) of 28 antimicrobial agents were determined using the broth microdilution method. The isolates were sequenced on an Illumina platform to identify antimicrobial resistance genes, virulence genes, and phylogenetic relationships.

**Results:**

The *S.* Welikade isolate (Sal097) was isolated from a two-year-old boy with acute gastroenteritis in 2021. Along with the other two isolates found in CLSSS, the three Chinese isolates were susceptible to all the examined antimicrobial agents, and their sequence types (STs) were ST5123 (*n* = 2) and ST3774 (*n* = 1). Single nucleotide polymorphism (SNP)-based phylogenetic analysis revealed that global *S.* Welikade strains can be divided into four groups, and these three Chinese isolates were assigned to B (*n* = 2; Sal097 and XXB1016) and C (*n* = 1; XXB700). In Group B, the two Chinese ST5123 isolates were closely clustered with three UK ST5123 isolates. In Group C, the Chinese isolate was closely related to the other 12 ST3774 isolates. The number of virulence genes in the *S.* Welikade isolates ranged from 59 to 152. The *galF* gene was only present in Group A, the *pipB2* gene was only absent from Group A, the *avrA* gene was only absent from Group B, and the *allB*, *sseK1*, *sspH2*, *STM0287*, and *tlde1* were found only within Group C and D isolates. There were 15 loci unique to the Sal097 isolate.

**Conclusion:**

This study is the first to characterize and investigate clinical *S.* Welikade isolates in China. Responsible for a pediatric case of gastroenteritis in 2021, the clinical isolate harbored no antimicrobial resistance and belonged to phylogenetic Group B of global *S.* Welikade genomes.

**Supplementary Information:**

The online version contains supplementary material available at 10.1186/s12864-024-10489-7.

## Background

*Salmonella* spp. is an important foodborne pathogen that was estimated to be responsible for 95.1 million cases and 50,771 deaths worldwide in 2017 [1]. *Salmonella* is composed of two recognized species: *Salmonella enterica* and *Salmonella bongori*, while *S. enterica* can be divided into 11 subspecies: *enterica* (I), *salamae* (II), *arizonae* (IIIa), *diarizonae* (IIIb), *houtenae* (IV), *indica* (VI), *londinensis* (VII), *brasiliensis* (VIII), *hibernicus* (IX), *essexiensis* (X), and reptilium (XI) [2]. According to the White-Kauffmann-Le Minor scheme, more than 2,600 serovars have been identified, among which more than half belonged to *S. enterica* subspecies *enterica* (1,586 serovars) [3].

Within *Salmonella enterica* subspecies *enterica*, the serovars Typhi (*S.* Typhi) and Paratyphi (*S.* Paratyphi) A, B, or C are responsible for enteric fever [4], while other serovars are collectively described as non-typhoidal *Salmonella* (NTS). In 2017, 14.3 million cases of enteric fever and 53.5 thousand cases of NTS invasive disease were estimated to occur globally [1, 5]. Sixteen serovars (*S. enterica* serovars Typhimurium, Enteritidis, Derby, London, Thompson, Agona, Rissen, Corvallis, Stanley, Kentucky, Weltevreden, Infantis, Newport, and Goldcoast) were responsible for approximately 80% of human *Salmonella* infections in China during 2006–2019 [6]. Among the remaining 20% of human salmonellosis cases, a few infections caused by uncommon serovars, such as *S.* Uzaramo [7], *S.* Wandsworth [8], *S.* Telelkebir [9], and *S.* Jangwani [10], have been reported. These uncommon serovars have been poorly described, and their clinical significance is always underappreciated, although they can cause invasive infections [7–9].

*S.* Welikade is an uncommon serovar that has not yet been reported in China, and even worldwide, it remains poorly described. There are a few reports citing *S.* Welikade strains isolated in Sri Lanka (1963) [11], Sweden (2003) [12], and Australia (2014) [13] and reports on sporadic cases and the outbreak in France (2022) [14]. However, no clinical data are available on the infections caused by this serovar. This lack of information might hinder clinicians from diagnosing *S.* Welikade infections in a timely manner and prevent patients from receiving proper treatments. The number of *S.* Welikade genomes is limited (approximately 50) in public genome databases, including NCBI and EnteroBase [15, 16].

The implementation of whole genome sequencing (WGS), which features the advantages of multilocus sequence typing (MLST) and pulse-field gel electrophoresis (PFGE) typing, has increased the understanding of *Salmonella* to a more comprehensive level. MLST is a sequence-based method, and an eBurst group (eBG) is composed of a central ST and its single-locus variants (SLVs) that correspond to a certain serovar in *S. enterica* subspecies *enterica*, such as eBG1, which corresponds to *S*. Typhimurium, and eBG4, which corresponds to* S*. Enteritidis [17]. MLST only characterizes seven housekeeping genes [18], while WGS can provide sequence data of all the genes carried by a strain for hierarchical analysis, from MLST to ribosomal MLST (rMLST), core genome MLST (cgMLST), and whole genome MLST (wgMLST). PFGE has been the golden standard for investigating foodborne outbreaks since the 2000s [19], with powerful discrimination but always focusing on one PFGE pattern that excludes closely related strains, while WGS provides greater discriminatory power and makes it possible to detect clusters with fewer cases than PFGE [20], which is especially suitable for investigating uncommon *Salmonella* serovars, including *S.* Welikade [14].

The aims of this study were to describe the first clinical case caused by the *S. enterica* serovar Welikade in China in 2021, to reveal the phenotype and genotype of the isolate, and to investigate the relationship of *S.* Welikade isolates from China and other counties by genomic analysis.

## Results

### *Salmonella* Welikade clinical isolate Sal097

We discovered a diarrhea case caused by a *Salmonella* Welikade (Sal097) isolate in Shanghai, China in August 2021. It was isolated from a two-year-old male child. The *Salmonella* isolate was serotyped by an agglutination assay at the Shanghai CDC and was confirmed to be an uncommon serovar, *Salmonella enterica* serovar Welikade (16:l,v:1,7). A retrospective investigation confirmed that the patient had no history of travel or pet contact during the month prior to the onset of diarrhea. Exposure factors, including a history of an unclean diet and contact with reptiles, were not found. No further investigations were performed on the food or living environment of the patient.

### Characterization of Chinese *Salmonella* Welikade isolates

For comparative purposes, another *S.* Welikade isolate (XXB1016, from a 17-year-old healthy carrier in 2013 in Guangxi) and another isolate of a monophasic variant of *S.* Welikade (XXB700, 16:l,v:-, from a 72-year-old male patient with diarrhea in 2013 in Shanghai) were included in the following investigations. XXB700 and XXB1016 were the only two *S.* Welikade isolates from a collection of more than 50,000 isolates in the CLSSS. Biochemical analysis performed using VITEK2 COMPACT revealed that all three isolates were in the *Salmonella* group (Supplementary Fig. 1), and the MALDI-TOF–MS results revealed that all three were *Salmonella enterica* ssp *enterica* (Supplementary Fig. 2). Antimicrobial susceptibility analysis revealed that the three Chinese isolates were susceptible to all 28 antimicrobial agents examined (Supplementary Table 1).

### Whole genome sequence analysis of the three Chinese *S.* Welikade isolates

The three Chinese *S.* Welikade isolates (Sal097, XXB700, and XXB1016) were subjected to Illumina sequencing. Based on the genome sequences, the sequence types (STs), antimicrobial resistance genes, and virulence genes were predicted. The STs of the three isolates were ST5123 (*n* = 2) and ST3774 (*n* = 1). Based on the ResFinder database, all three isolates were predicted to harbor the *aac(6’)-Iaa* gene, and no other antimicrobial resistance genes were found. Based on the VFDB database, the three isolates were predicted to carry 121–145 virulence genes (Supplementary Table 2).

### Phylogenetic analysis of *Salmonella* Welikade

To place the Chinese isolates into the context of global *S.* Welikade strains, we collected genome sequence data from GenBank and EnteroBase (accessed 31 November 2023), and found 53 additional *S.* Welikade isolates (including two isolates from China in 2016) and 16 *S.* Welikade monophasic variant (16:l,v:-) isolates from various sources and from different continents. All 72 genomes were evaluated to be qualified using CheckM, with completeness ranging from 97.41% to 99.68% and contamination ranging from 0.31% to 1.76% (Supplementary Table 3).

**Fig. 1:**
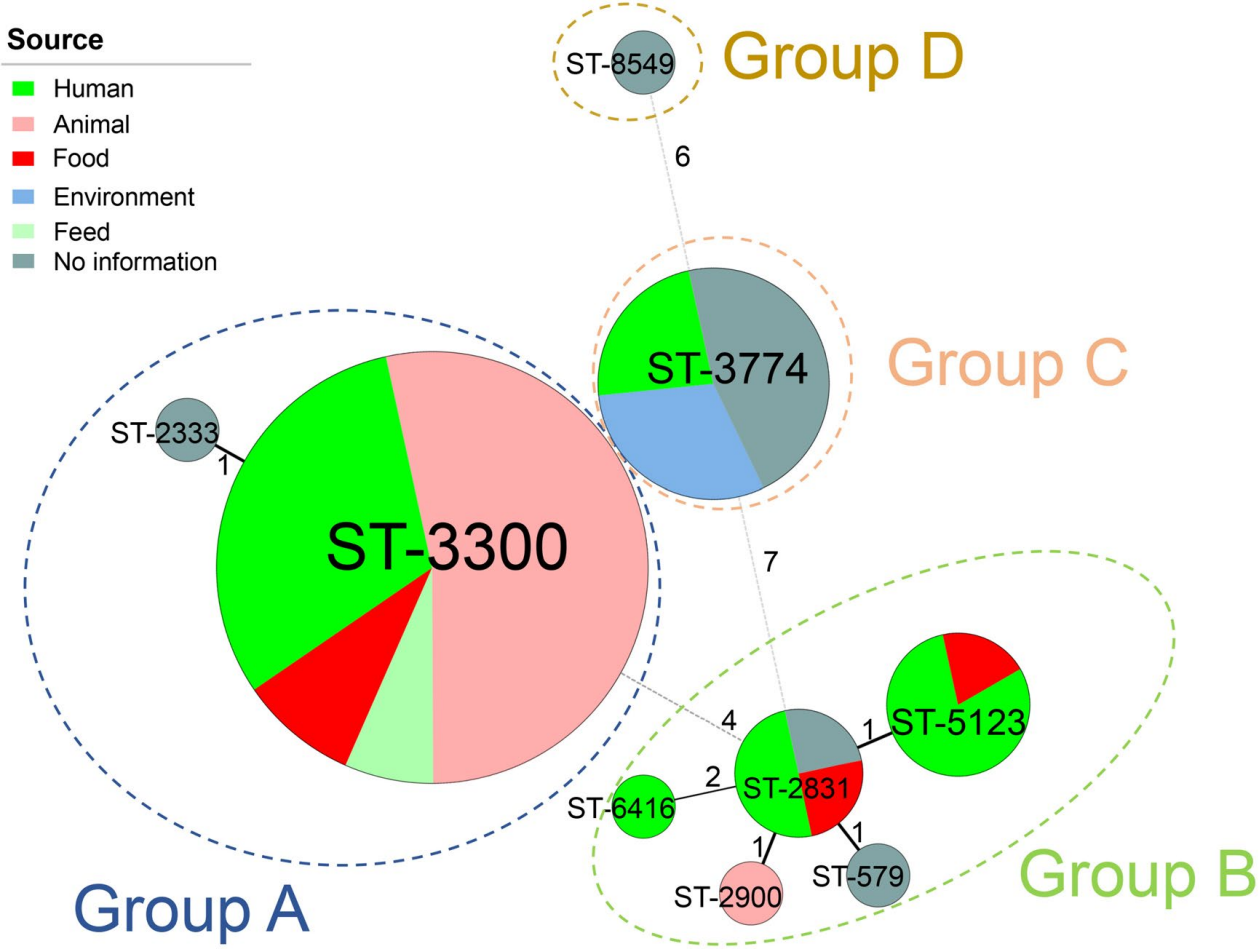
Minimum spinning tree analysis of the multilocus sequence type (MLST) data of 72 *S.*Welikade isolates. Each ST is displayed as a circle, and the size of the circle indicates the number of isolates of this ST. Dsashed lines surround the STs that belong to the same groups, and the numbers of different loci between neighboring STs are indicated

**Fig. 2:**
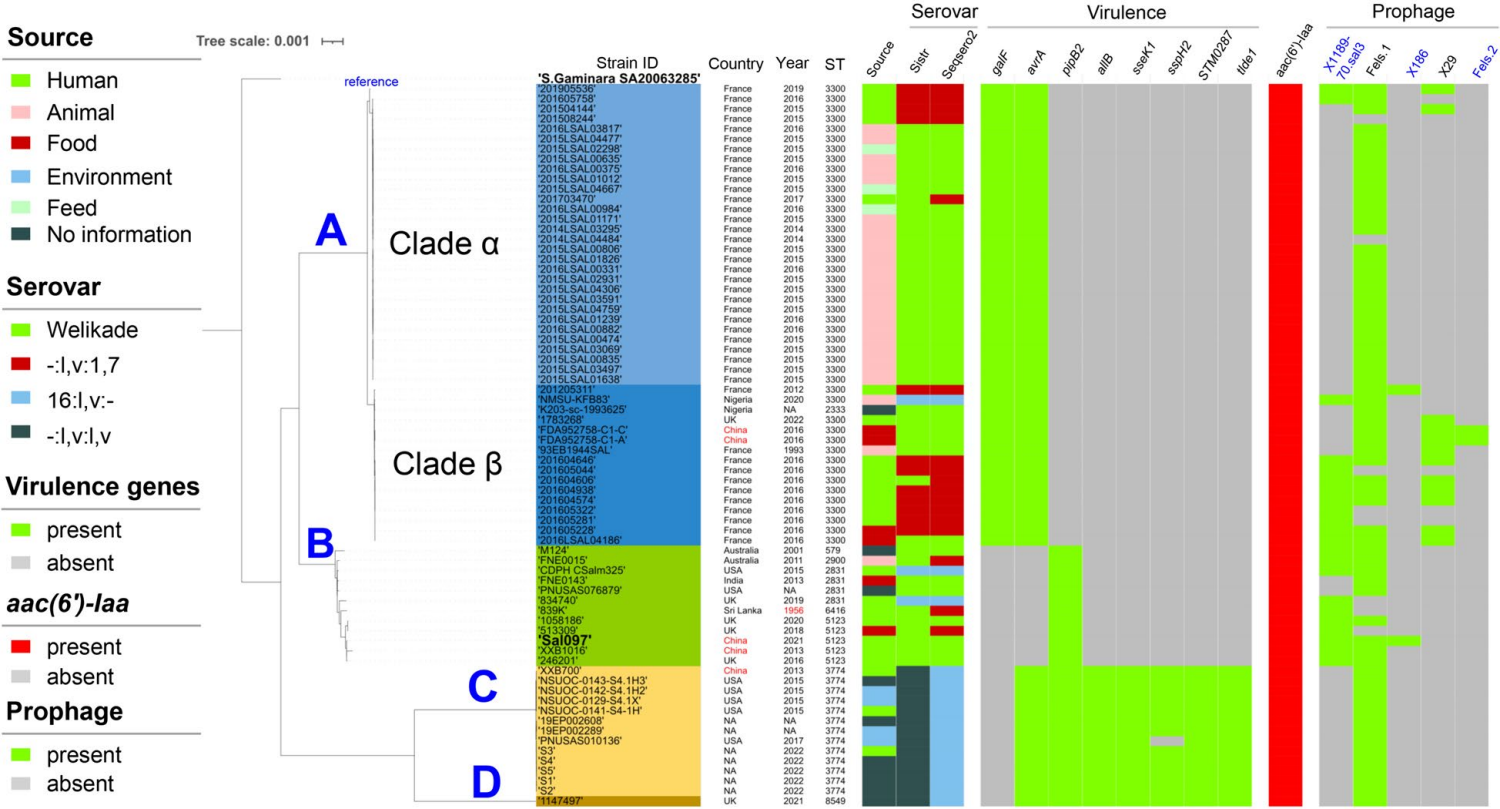
Phylogenetic analysis based on the core‑genome SNPs of *S.* Welikade isolates. The genome of *S.* Gaminara SA20063285 was used as outgroup

The serovars of the 72 genomes were checked based on the genome sequences (Supplementary Table 3). According to the SISTR results, the strains were assigned to three serovars, namely, I:l,v:l,v (*n* = 49), Z:l,v:l,v (*n* = 12), and Welikade (*n* = 11). Using SeqSero2, the serovars of the 72 genomes were predicted to be Welikade (*n* = 38), I 16:l,v:- (*n* = 17), and I -:l,v:1,7 (*n* = 17). Using mlst software, the 72 genomes were found to represent nine STs, with ST3300 (*n* = 45) being predominant, followed by ST3774 (*n* = 13) (Supplementary Table 4). Minimum spinning tree analysis of these STs revealed four groups (Fig. 1). SNP-based phylogenetic analysis revealed that global *S.* Welikade strains can be divided into four groups, A (*n* = 46), B (*n* = 12), C (*n* = 13), and D (*n* = 1) (Fig. 2). Group A was composed of isolates assigned to ST3300 (*n* = 45) and its single-locus variant (SLV; ST2333, *n* = 1) (Fig. 1), and can be further divided into two clades (*α* and *β*) as described by Cherchame et al. [14]. Group B was composed of isolates assigned to ST2831 (*n* = 4) and its SLVs (ST579, *n* = 1; ST2900, *n* = 1; ST5123, *n* = 5) or its double-locus variants (DLVs; ST6416, *n* = 1). The first *S.* Welikade isolate worldwide, 839 K (1956, Sri Lanka), was also included in the Group B. Groups C and D were both composed of isolates with serovars predicted to be monophasic variants of *S.* Welikade. Group C isolates all belonged to ST3774, while Group D isolates belonged to ST8549.

There were five Chinese genomes, including two from food deposited in the EnteroBase database. Assigned to Group B, Sal097 and XXB1016 were closely related to three UK isolates (246201, 513309, and 1058186), and all five of these isolates belonged to ST5123. XXB1016 was most closely related to another ST5123 isolate, 246201, which was isolated from a British individual in 2016, and both of these strains were adjacent to Sal097 (Fig. 2). XXB700 was assigned to Group C and clustered closely with the other 12 ST3774 isolates. The two Chinese isolates in the public database (FDA952758-C1-A and FDA952758-C1-C) were very closely related and were assigned to Group A clade *β*, both with ST3300.

### Analysis of antimicrobial resistance genes in the *Salmonella* Welikade population

To determine whether the antimicrobial resistance genes of the *S.* Welikade isolates from China and from other countries differ, we used the ResFinder database to analyze all the 72 genomes. WGS analysis indicated that all the *S.* Welikade isolates carried only one antimicrobial resistance gene, *aac(6’)-Iaa*, encoding an aminoglycoside N-acetyltransferase (Supplementary Table 5).

### Prophage analysis of the *Salmonella* Welikade population

Prophage analysis using PHASTER revealed that the number of prophages in each *S.* Welikade genome ranged from one to five (Supplementary Table 6). Most of the *S.* Welikade isolates carried the *Salmonella* phage Fels-1 (NCBI NC_010391), and isolates of different phylogenetic groups had some distinct prophages (Fig. 2). Group A cluster *α* was linked to phages SI7, SEN34, and SW9; Group A cluster *β* was linked to phages 118970_sal3 (NC_031940), Entero_186 (NC_001317), and Entero_fiAA91_ss (NC_022750); and Group B was linked to phage 118970_sal3 (NC_031940). All these prophages carry no antimicrobial resistance genes. No isolates carried the same prophage profile as did the Sal097 strain (Fels-1, 118970_sal3, and Entero_186).

### Virulence genes in the *Salmonella* Welikade population

Analysis through the VFDB database indicated that the number of virulence genes among the *S.* Welikade genomes ranged from 59 to 152. These virulence genes were associated with the type III secretion system (TTSS; *n* = 81), adherence (*n* = 25), the effector delivery system (*n* = 22), the type VI secretion system (*n* = 81), and other functions (*n* = 23) (Supplementary Table 2). The gene *galF* (capsule-associated) was only present in Group A, while the gene *pipB2* (TTSS secreted effectors) was absent from Group A isolates and was present in the other three groups. The gene *avrA* (part of the *Salmonella* Pathogenic Island-1 [SPI-1]) was absent from group B isolates but was present in isolates of the other three groups. The genes *allB* (nutritional/metabolic factor), *sseK1* (TTSS-2 secreted effector), *sspH2* (TTSS-2 secreted effector), *STM0287* (effector delivery system), and *tlde1* (effector delivery system) were found only within the Group C and D isolates.

Among the five ST5123 genomes, Sal097, 513309, and 1058186 harbored 122 virulence genes while both XXB1016 and 246201 harbored 121 virulence genes, differing only in an uncommon virulence gene, *gogB*, which was only present in four Group B isolates. The *gogB* gene encodes the TTSS effector GogB, an anti-inflammatory effector. XXB700 harbored 145 virulence genes, which were shared by almost all the Group C isolates.

### Unique loci of Chinese *Salmonella* Welikade

To determine whether the Chinese *S.* Welikade isolates possess any unique loci, we performed pan-genome analysis on the 72 *S.* Welikade genomes. Roary analysis revealed 3,768 core genes (present in > 95% of the genomes) in the *S.* Welikade genome and 3,744 accessory genes. There were 15 loci unique to the Sal097 strain, including two genes encoding helix-turn-helix domain-containing proteins, one gene encoding the chromosome-partitioning ATPase Soj, one gene encoding the host-nuclease inhibitor protein Gam, and one gene encoding the DUF4747 family protein (Table 1). There were seven loci unique to the isolate XXB1016, including the gene *fimA* encoding the type-1 fimbrial protein A chain and one gene encoding the DUF4222 domain-containing protein. There were no loci unique to the XXB700 strain.

**Table 1:** Unique genes of Chinese *Salmonella* Welikade isolates^a^

No	Locus	Gene	Annotation	Size (bp)	Unique to which isolate	GenBank Accession number
1	Sal097_01762	NA^b^	hypothetical protein	414	Sal097	OQ207679
2	Sal097_01773	NA	hypothetical protein	633	Sal097	OQ207680
3	Sal097_01774	NA	hypothetical protein	486	Sal097	OQ207681
4	Sal097_01781	NA	helix-turn-helix domain-containing protein	243	Sal097	OQ207682
5	Sal097_01782	NA	helix-turn-helix domain-containing protein	396	Sal097	OQ207683
6	Sal097_01783	*soj*	chromosome-partitioning ATPase Soj	840	Sal097	OQ207684
7	Sal097_01784	NA	hypothetical protein	387	Sal097	OQ207685
8	Sal097_01785	NA	hypothetical protein	663	Sal097	OQ207686
9	Sal097_01787	NA	host-nuclease inhibitor protein Gam	297	Sal097	OQ207687
10	Sal097_02625	NA	hypothetical protein	1326	Sal097	OQ207688
11	Sal097_02628	NA	hypothetical protein	276	Sal097	OQ207689
12	Sal097_02638	NA	hypothetical protein	798	Sal097	OQ207690
13	Sal097_03127	NA	DUF4747 family protein	894	Sal097	OQ207691
14	Sal097_03128	NA	hypothetical protein	606	Sal097	OQ207692
15	Sal097_04349	NA	hypothetical protein	213	Sal097	OQ207693
16	XXB1016_01434	NA	hypothetical protein	1461	XXB1016	OQ207694
17	XXB1016_01703	*fimA*	type-1 fimbrial protein, A chain	585	XXB1016	OQ207695
18	XXB1016_01709	NA	hypothetical protein	222	XXB1016	OQ207696
19	XXB1016_02949	NA	DUF4222 domain-containing protein	180	XXB1016	OQ207697
20	XXB1016_02953	NA	hypothetical protein	894	XXB1016	OQ207698
21	XXB1016_02955	NA	hypothetical protein	309	XXB1016	OQ207699
22	XXB1016_02956	NA	hypothetical protein	339	XXB1016	OQ207700

^a^The unique genes were discovered through the Roary analysis

^b^*NA* Not applicable

## Discussion

The *Salmonella* Welikade isolates detected in this study is a relatively rare serovar (3 of > 50,000 Chinese *Salmonella* isolates and 72 of 464,493 *Salmonella* isolates deposited in the EnteroBase database) and this study is the first to characterize and investigate clinical *S.* Welikade isolates in China. The clinical strain isolated in 2021 was responsible for a pediatric case of gastroenteritis, harbored no antimicrobial resistance and belonged to phylogenetic Group B of global *S.* Welikade genomes, which was mainly composed of ST5123 and ST2831 isolates.

Because no data are available on *S.* Welikade clinical isolates in China, we searched reports from other countries and found no detailed descriptions of clinical infections caused by this serovar. According to previous studies, *S.* Welikade clinical isolates were reported in Australia from 1985 to 2000 (*n* = 144), mostly from patients with intestinal complaints [13], and in France, *S.* Welikade isolates were responsible for an outbreak involving eight patients in 2016 and for five sporadic cases from 2012 to 2019 [14], without describing on the infections of these 13 patients. Other sources of *S.* Welikade isolates include poultry (laying hens and broilers), food (desiccated coconut, blanched peanut kernel, goat cheese, and spice mix), wildlife (lizard, snake, tropical rock lobster, and gull), feed, and the environment (soil/dust) [11–14]. The case in this study involved sporadic gastroenteritis in a toddler, and the duration of disease was less than one week. Unfortunately, no food, pet, or living environment specimens associated with the patient were available for further investigation. However, by WGS analysis, we connected this isolate with four other ST5123 *S.* Welikade isolates, including an isolate collected in a distant province in China 8 years ago and three isolates from the UK (Fig. 2). The five ST5123 isolates, isolated from humans (*n* = 4) and food (*n* = 1) closely clustered together and might have originated from the earliest *S*. Welikade isolate (839 K) from Sri Lanka in 1956. Our study provides an example of the use of public genomes of the same serovar to investigate a clinical case involving by *Salmonella* isolates of a rare serovar, which has extended our knowledge on the diversity, potential origin, and genetic characteristics of the virulence and antimicrobial resistance of an unusual cause.

Combined with the genomes of the monophasic variant of *S.* Welikade, we found that the global *S.* Welikade genomes could be divided into four groups, each of which was associated with a different central ST (Figs. 1 and 2). All the central STs differed by at least 4 of the 7 loci, which suggests that the population structure of *S.* Welikade is polyphyletic, similar to that of *S*. Newport rather than *S*. Typhi. Multiple and discrete lineages have been identified within serovar Newport [21]; in contrast, *S*. Typhi is genetically monomorphic [22]. Over 60% (46/72) of the *S.* Welikade genomes were assigned to Group A, with other groups each accounting for less than 20%; this structure is similar to the population structures of *S*. Typhimurium and *S*. Enteritidis in that most isolates were in one primary eBG and rare isolates were present in multiple unrelated eBGs and STs [17]. According to the serovars predicted by SISTR and SeqSero2, 17 isolates (16:l,v:-) might be monophasic variants of *S*. Welikade. Except for 3 isolates assigned to Groups A and B, the other 14 isolates were far from the *S*. Welikade genomes in the phylogenetic tree (Fig. 2), which suggests that these 14 isolates might have evolved from a different lineage of *S*. Welikade. Similarly, STs of these 14 isolates were distant from the STs of the *S*. Welikade isolates (Fig. 1).

SISTR and SeqSero2 are widely used for the prediction of *Salmonella* serovars [23, 24]. However, in this study, the results provided by these two software programs were not satisfactory. Among the 53 isolates with serum agglutination results similar to that of *S*. Welikade, the accuracy rates of SISTR and SeqSero2 were 17.0% (9/53) and 67.9% (36/53), respectively (Supplementary Table 3). The eBurst groups (eBG) based on MLST have been used to correspond to certain serovars in *S. enterica* subspecies *enterica* [17], but to date, no eBG has been defined to correspond to *S.* Welikade. Based on our analysis and a previous report [14], ST3300 (central ST of Group A) and ST2831 (central ST of Group B) with their respective SLVs can constitute two distinct eBGs corresponding to *S.* Welikade, while ST3774 (central ST of Group C) could be a primary representative of monophasic variants of *S*. Welikade.

Five Chinese *S.* Welikade genomes were deposited in the open database, and they were assigned to three phylogenetic groups (A, B, and C). Although the genomes of the three phylogenetic groups each harbored more than 50 virulence genes, some genes were specific to certain groups. The virulence gene *galF* was unique to Group A, including the two Chinese *S*. Welikade isolates from food (blanched peanut kernel); this gene encodes the UTP–glucose-1-phosphate uridylyltransferase GalF, which is responsible for the translocation and surface assembly of capsular polysaccharides [25]. The genes *allB*, *sseK1*, *sspH2*, *STM0287*, and *tlde1* were unique to Groups C and D, including the Chinese *S*. Welikade isolate XXB700 (Fig. 2). The *allB* gene encodes allantoinase, which is involved in the allantoin utilization of *Salmonella enterica* [26]. The *sseK1* gene encodes the glycosyltransferase SseK1, which can glycosylate host proteins on arginine and increase the glycolysis in macrophages infected by S. Typhimurium by down-regulating the inflammation-related cytokines [27]. The *sspH2* gene encodes the novel E3 ubiquitin-ligase SspH2, which can specifically localize to the brush border of polarized epithelial cells [28]. The *STM0287* gene encodes a putative periplasmic protein and its function remains unclear [29]. The *tlde1* gene encodes the type VI secretion system effector Tlde1, which displays both _L,D_-carboxypeptidase activity by cleaving peptidoglycan tetrapeptides between meso-diaminopimelic acid^3^ and _D_-alanine^4^ and _L,D_-transpeptidase exchange activity by replacing _D_-alanine^4^ with a non-canonical _D_-amino acid [30]. Although no virulence genes were found to be unique to Group B, which contained the Chinese isolates Sal097 and XXB1016, these two isolates each had a few unique loci (Table 1). Among the 15 loci unique to Sal097, the function of the *soj* gene is relatively clear. In *Bacillus subtilis*, Soj regulates DNA replication initiation and is involved in cell cycle changes during endospore formation [31]. Among the seven loci unique to XXB1016, the function of the *fimA* genein *Salmonella* has been studied. *S.* Typhimurium type 1 fimbrial proteins are encoded by the *fim* gene cluster, and FimA is the major subunit [32].

The screening of antimicrobial resistance-associated genes, revealed that all the *S*. Welikade genomes harbored only the *aac(6’)-Iaa* gene, which encodes an aminoglycoside N-acetyltransferase. However, no antimicrobial resistance, including resistance to aminoglycosides, was detected phenotypically (Supplementary Table 1). This is not unusual because several studies have reported low-aminoglycoside MIC values in *aac(6’)-Iaa*-harboring Enterobacterales [33, 34]. The presence of the *aac(6’)-Iaa* gene does not necessarily confer phenotypic resistance to aminoglycosides, as it is often weakly expressed or not expressed at all [35].

One limitation of this study is that we did not obtain food, pet, or living environment specimens associated with the patient in Shanghai in 2021; thus we were unable to investigate the source of the *S*. Welikade isolate (Sal097). However, we linked this isolate with another Chinese *S*. Welikade isolate (XXB1016) by phylogenetic analysis.

## Conclusions

In conclusion, for the first time, we reported a clinical case caused by the *S. enterica* serovar Welikade in China, and the isolate exhibited no antimicrobial resistance. There are four phylogenetic groups among the global *S.* Welikade genomes, and this clinical case was caused by an isolate of Group B. The isolate was closely clustered with other ST-5123 isolates from humans and food. Although infections caused by *S.* Welikade are uncommon, there is a need to collect data on this serovar globally to allow a more comprehensive characterization.

## Materials and methods

### Clinical investigation

A 2-year-old boy with no significant medical history visited the outpatient department of a tertiary hospital with a two-day history of fever and diarrhea in Shanghai on August 23, 2021. The patient had no history of travel or contact with any wildlife or domestic animals. In the last two days, the patient had diarrhea 5 or 6 times per day, without pus or bloody stool. The patient also complained of vomiting after meals. Laboratory investigation indicated an elevated fecal leukocyte count (30 per high power field), an increased fecal red-blood-cell count (40 per high power field), and a positive fecal occult blood test. Blood tests revealed a white blood cell (WBC) count of 7.7 × 10^9^/L with 57.9% neutrophils and a C-reactive protein (CRP) level of 18.1 mg/L. Stool samples were collected for cultures of *Salmonella*, *Shigella*, and *Vibrio parahaemolyticus*, and human rotavirus antigen and adenovirus antibody (IgM) assays were performed. The culture for *Salmonella* was positive, as was the assay for human rotavirus antigen. Then, the *Salmonella* isolate was sent to the Shanghai Center for Disease Prevention and Control (Shanghai CDC) for further study. The clinical diagnosis was acute gastroenteritis and cefixime capsules were administered as an empirical treatment. The patient recovered after three days of treatment.

### Characterization of Chinese *Salmonella* Welikade isolates

Stool samples were cultured on the second day after onset, and positive colonies were detected on xylose-lysine-deoxycholate (XLD) agar plates (Supplementary Fig. 3). The isolated bacterium (Sal097) was subjected to biochemical analysis (VITEK2 COMPACT; bioMérieux) and was identified as a member of the *Salmonella* group (Supplementary Fig. 1). Pure bacterial colonies were spotted on the MALDI biotarget and cocrystallized with the MSCHAC matrix. Spectra were acquired with a matrix-assisted laser desorption/ionization time-of-flight mass spectrometry (MALDI-TOF–MS; bioMérieux), and the result obtained indicated *Salmonella enterica* ssp *enterica* (Supplementary Fig. 2). A serotype agglutination assay was conducted using a commercial *Salmonella* antiserum kit (SSI Diagnostica, Denmark), which confirmed the isolate to a member of an uncommon serovar (Welikade [16:l,v:1,7]) in China.

For comparative purposes, we also investigated additional *S.* Welikade isolates in the Chinese Local Surveillance System for *Salmonella* (CLSSS). The CLSSS, which is led by the Shanghai Municipal Centre for Disease Prevention and Control (CDC), has deposited over 50,000 *Salmonella* isolates from human, animal, and environmental samples over recent decades from the CDCs of 20 provinces or municipal cities in China [5]. In addition to the isolate Sal097, we discovered two other *S.* Welikade isolates in China. Both strains were isolated in 2013. One *S.* Welikade isolate (XXB700) was isolated from the stool of a 72-year-old male patient with sporadic diarrhea in Shanghai. The other isolate (XXB1016) was isolated from the stool of a healthy 17-year-old boy in Guangxi Province, which is approximately 2,000 km from Shanghai. The serovar of the isolate XXB1016 was 16:l,v:-, which seemed to be a monophasic variant of S. Welikade. All the confirmed *Salmonella* isolates were subjected to a serological agglutination assay according to the White-Kauffmann-Le Minor classification scheme (SSI Diagnostica) [3]. Therefore, we included only the three available Welikade isolates (Sal097, XXB700, and XXB1016) in the following biological investigations.

### Antimicrobial susceptibility test

The minimum inhibitory concentrations (MICs) of 28 antimicrobial agents were determined and interpreted with the broth microdilution method procedure recommended by the guidelines of the Clinical and Laboratory Standards Institute (CLSI) in 2023 [36]. The MICs (μg/m1) of the 28 agents (Fosun Pharma, Shanghai) used in our assay were as follows: ampicillin (1–64), ampicillin/sulbactam (1–64), colistin E (0.125–8), cefazolin (0.5–32), cefotaxime (0.25–16), ceftazidime/clavulic acid (0.25/4–16/4), cefotaxime/clavulic acid (0.125/4–8/4), cefoxitin (1–64), cefepime (1–64), cefuroxime (0.5–32), ceftazidime/avibactam (0.25/4–8/4), chloramphenicol (2–64), trimethoprim-sulfamethoxazole (0.25/4.75–8/152), aztreonam (2–64), imipenem (0.25–8), ertapenem (0.25–8), meropenem (0.125–4), ceftazidime (0.25–32), tetracycline (1–32), tigecycline (0.25–8), ciprofloxacin (0.015–32), levofloxacin (0.125–4), norfloxacin (0.125–64), nalidixic (2–64), azithromycin (2–64), amikacin (2–64), streptomycin (4–32), and gentamicin (1–32). *Escherichia coli* ATCC25922, *E. coli* ATCC35218, *Staphylococcus aureus* ATCC29213, and *Enterococcus faecalis* ATCC29212 were used as reference strains.

### Genomic sequencing and data analysis

After overnight growth on Columbia agar (Oxoid, Basingstoke, United Kingdom) supplemented with 5% sheep blood, bacteria were removed with an inoculation loop suspended in 1 mL of PBS, and pelleted by centrifugation for 10 min at 5000 g. The bacterial pellet was resuspended in 180 µL of Buffer ATL, after which the genomic DNA samples of the Sal097, XXB700, and XXB1016 strains were purified using a Qiagen DNA Mini Kit (Qiagen, Hilden, Germany) according to the manufacturer’s protocol. Genome sequencing was performed on an Illumina NextSeq platform using a paired-end strategy with a 150-base read length. The average coverage depth of the genomes was approximately 220-fold. The sequence rawdata were assembled using the SPAdes (version 3.15.5) algorithm and filtered with a depth of 10 and a scaffold length of 200 bp [37]. In addition to these three Chinese isolates, we searched the *S.* Welikade genome in the open databases GenBank and EnteroBase (accessed 31 November 2023) and collected 69 additional *S.* Welikade isolates, including 16 *S.* Welikade monophasic variant (16:l,v:-) isolates from various sources and from different continents (Supplementary Table 3). The completeness and contamination of the genomes were evaluated using CheckM [38], with the cutoff values of > 95% completeness and < 5% contamination.

All the qualified genomes were screened for antimicrobial resistance genes and virulence factors using ResFinder (updated on Dec 23, 2023) and the Virulence Factors Database (VFDB, updated on Dec 23, 2023) [39, 40], respectively. The serovar of the *Salmonella* genomes was predicted using the *Salmonella* In Silico Typing Resource (SISTR, version 1.1.1) platform and SeqSero2 (version 1.2.1) [23, 24]. Multilocus sequence typing (MLST) of the genomes was performed using mlst (version 2.11; https://github.com/tseemann/mlst) [41], and the obtained sequence types (STs) were searched in EnteroBase to determine whether the corresponding serotypes and eBurst groups (eBGs) were available [17, 42]. Minimum spanning tree analysis of the MLST data was performed using the BioNumerics software package (version 6.5; Applied Maths). The prediction of prophages was performed using PHASTER [43]. To determine the population structure of the 72 available *S.* Welikade genomes, the complete genome of *S.* Gaminara SA20063285 (NCBI accession number: NZ_CP030288.1) was used as the outgroup genome in accordance with a previous study [14], and single-nucleotide polymorphism (SNP) phylogenetic core-genome analysis was performed on the 72 *S.* Welikade genomes using the Parsnp (version 1.2) pipeline [44].

### *Pan*-genome analysis

To determine the unique loci present in a certain isolate but absent from other isolates among the Chinese *S.* Welikade isolates, pan-genome analysis was performed. The 72 genomes were annotated by Prokka (version 1.14.6) [45] to acquire gff files, which were input into the Roary (version 3.13) pipeline to discover the accessory loci [46]. The minimum percentage identity for BLASTP was set as 90%.

### Supplementary Information

Supplementary Materials 1.

Supplementary Materials 2.

## Data Availability

The genome sequence of the isolate Sal097 was deposited in NCBI (BioSample SAMN32538455), and the whole genome shotgun project was deposited at DDBJ/ENA/GenBank under the accession JAQGEG000000000. The XXB700 and XXB1016 genomes were assigned as BioSample Nos. of SAMN21890787 and SAMN21889512, respectively. The associated metadata and virulence genes can be found in Supplementary Tables 1 to 6. The sequences of the unique loci of Sal097 and XXB1016 were submitted to GenBank under accession numbers OQ207679 to OQ207700.
