# Supplementary material for: Genomic investigation of Salmonella enterica Serovar Welikade from a pediatric diarrhea case first time in Shanghai, China
Source: BMC Genomics. 2024 Jun 17;25:604. doi: 10.1186/s12864-024-10489-7 (PMC11181664; doi:10.1186/s12864-024-10489-7)
Supplement: Supplementary file 2 — Supplementary Materials 2. [file 12864_2024_10489_MOESM2_ESM.docx]

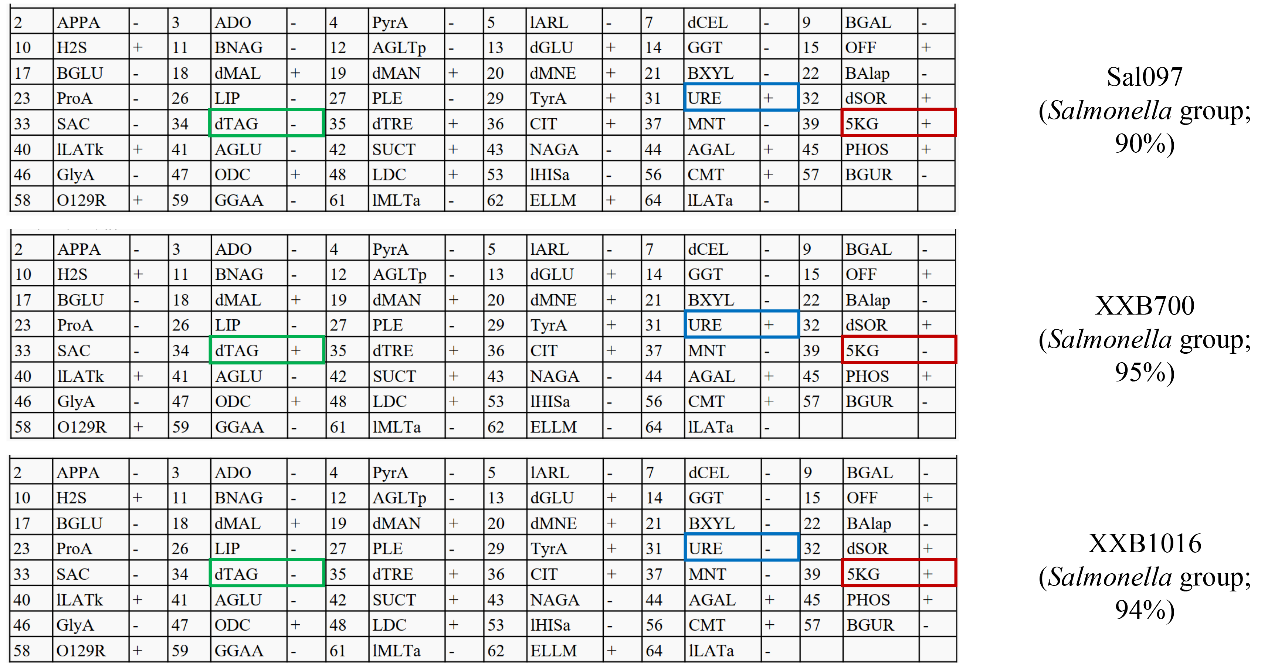


**Supplementary Figure 1. Biochemical results of the three Chinese *Salmonella* Welikade isolates tested using VITEK2 COMPACT.** The biochemical tests showing different results are labelled with box.

**
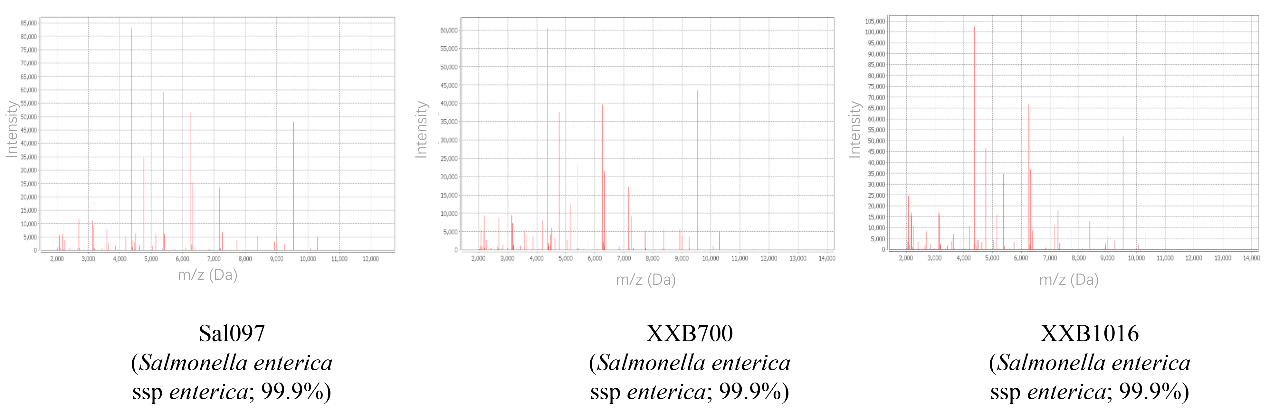
**

**Supplementary Figure 2. Identification results of the three Chinese *Salmonella* Welikade isolates tested using MALDI-TOF-MS.**

**
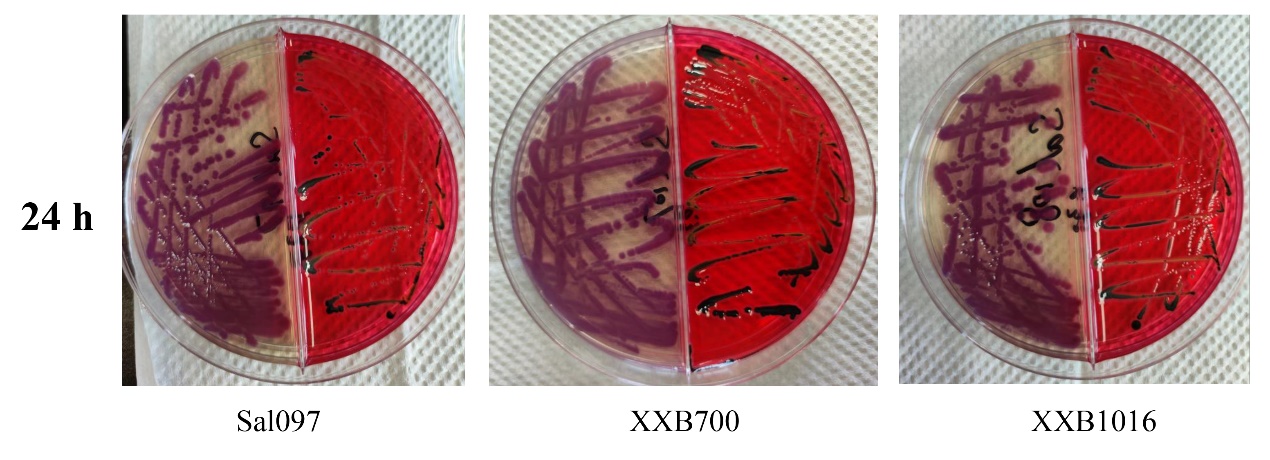
**

**Supplementary Figure 3. Colony morphology of the three Chinese *Salmonella* Welikade isolates on the chromogenic media.** The chromogenic medium is composed of two kinds of media. The left is chromogenic *Salmonella* agar base. When *Salmonella* is cultured on this media, the colonies will show wine red. The right is xylose lysine desoxycholate (XLD) medium. When *Salmonella* is cultured on this media, the colonies will show black in center.
